# Chikungunya virus capsid protein contains nuclear import and export signals

**DOI:** 10.1186/1743-422X-10-269

**Published:** 2013-08-28

**Authors:** Saijo Thomas, Jagdish Rai, Lijo John, Stephan Schaefer, Brigitte M Pützer, Ottmar Herchenröder

**Affiliations:** 1 Institute of Experimental Gene Therapy and Cancer Research, Rostock University Medical Center, Schillingallee 69, 18057, Rostock, Germany; 2 Department of Molecular Biology and Bioinformatics, Tripura University Suryamaninagar, Tripura 799022, India; 3 Current address: School of Biological Sciences, Monash University, Clayton, VIC 3800, Australia; 4 Current address: MCDB, University of California, Santa Barbara, CA 93106-9625, USA; 5 Current address: LADR GmbH Medizinisches Versorgungszentrum Mecklenburg-Vorpommern, Rostock, Germany

**Keywords:** Chikungunya virus, Capsid protein, Nuclear transport, Nuclear localization signal, Nuclear export signal, Karyopherin, CRM1

## Abstract

**Background:**

Chikungunya virus (CHIKV) is an *alphavirus* of the *Togaviridae* family. After autoproteolytic cleavage, the CHIKV capsid protein (CP) is involved in RNA binding and assembly of the viral particle. The monomeric CP is approximately 30 kDa in size and is small enough for passive transport through nuclear pores. Some *alphavirus*es are found to harbor nuclear localization signals (NLS) and transport of these proteins between cellular compartments was shown to be energy dependent. The active nuclear import of cytoplasmic proteins is mediated by karyopherins and their export by exportins. As nuclear and cytoplasmic trafficking may play a role in the life cycle of CHIKV, we have sought to identify nuclear localization and nuclear export signals in CHIKV CP in a virus-free system.

**Methods:**

EGFP-fusion proteins of CHIKV CP and mutants thereof were created and used to monitor their intracellular localization. Binding of cellular proteins was confirmed in pull-down assays with purified CP using co-immuoprecipitation. Nuclear localization was demonstrated in a virus-free system using fluorescence microscopy.

**Results:**

Here we show that CHIKV CP is a nuclear-cytoplasmic shuttling protein with an active NLS that binds to karyopherin α (Karα) for its nuclear translocation. We also found that the Karα4 C-terminal NLS binding site is sufficient for this interaction. We further demonstrate that CHIKV CP interacts directly with the export receptor CRM1 to transport this viral protein out of the nucleus via a nuclear export signal (NES). The CHIKV CP NES was mapped between amino acids 143 and 155 of CP. Deduced from *in silico* analyses we found that the NES has a mode of binding similar to the snurportin-1 CRM1 complex.

**Conclusions:**

We were able to show that in a virus-free system that the CHIKV capsid protein contains both, a NLS and a NES, and that it is actively transported between the cytoplasma and the nucleus. We conclude that CHIKV CP has the ability to shuttle via interaction with karyopherins for its nuclear import and, *vice versa*, by CRM1-dependent nuclear export.

## Background

The arthropode-borne Chikungunya virus (CHIKV) of the genus *alphavirus* in the *Togaviridae* family is running rampant along the Western and Northern Rim of the Indian Ocean. Outbreaks occurred on its islands including the Malay Archipelago as well as in several tropical African countries [1-3]. *Alphaviruses* express a capsid protein (CP) as part of a polyprotein which, after autoproteolytic cleavage by its protease activity, forms viral particles and packages the viral genomic RNA [3]. Although being small enough (approx. 30 kDa) for passive transport through nuclear pores, some *alphaviral* capsid proteins are reported to harbor a nuclear localization signal (NLS) with the protein’s transport being energy dependent [4,5].

In general, bidirectional transport of molecules between the nucleus and the cytoplasm occurs through the nuclear pore complex (NPC), a supra-molecular structure of the nuclear envelope [6]. NPCs allow passive diffusion of ions and small proteins up to a molecular weight of 40 kDa or less than 9 nm in diameter, but restrict passage of larger molecules to those containing an appropriate targeting signal [7]. Protein traffic into the nucleus is mediated by the interaction of transport cargoes with karyopherins. Karyopherins are adaptor proteins that recognize cargo to be conveyed via a NLS which also interacts with the transport receptor importin β. Together, these proteins form a ternary bundle that conjointly translocates into the nucleus via the nuclear pore complex [8]. NLS motifs used by the classical nuclear import pathway consist of a short stretch of the positively charged amino acids (aa) arginine and lysine but lack strict consensus sequences [9]. A monopartite NLS like that of SV40 large T-antigen is composed of a cluster of five to seven basic aa, while a typical bipartite NLS contains two clusters of basic amino acids separated by a linker of 10–11 aa [10-12]. Nuclear export signals (NES) are recognized by exportins and allow proteins to be actively carried from the nucleus to the cytoplasm through the NPC [13]. NES are specifically bound by exportin known as exportin 1 or Chromosomal Region Maintenance 1 (CRM1). This transport is mediated via binding to the GTP-bound form of the guanine nucleotide-binding protein Ran. The most commonly identified NES are short leucine-rich stretches, although other hydrophobic residues such as isoleucine, methionine, phenylalanine, and valine can also contribute to this entity [14].

In the present study, we searched for motifs in the CHIKV CP that would enable this protein to shuttle between cellular compartments. As nuclear and cytoplasmic trafficking of the viral CP may also play a role in the CHIKV life cycle, we have sought to identify a NLS and, consequently, looked for a nuclear export signal (NES) in the CHIKV CP. Here, we demonstrate that CHIKV CP binds to karyopherin α (Karα) for its nuclear translocation, and that the Karα4 C-terminal NLS binding domain is sufficient for this interaction. We also show that CHIKV CP contains a CRM1-mediated NES, which is mapped to a leucine rich region between amino acids 143 and 155 and docks similar to snurportin-1 CRM1 complex *in silico*. Applying virus-free methods, we demonstrate that CHIKV CP contains motifs enabling it to actively shuttle between the cytoplasm and the nucleus.

## Results

### The CHIKV CP contains an active NLS and binds to Karα4

**Figure 1:**
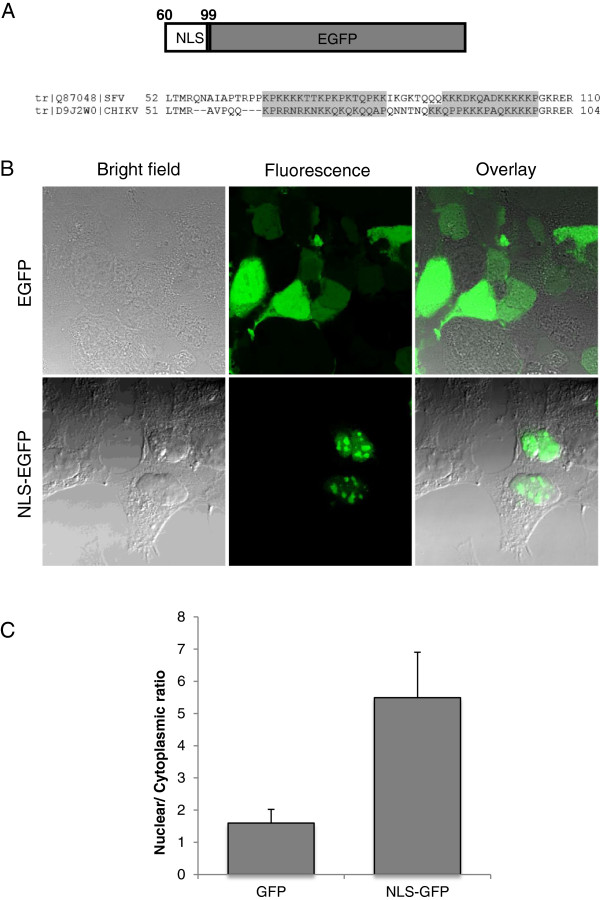
**CHIKV CP contains an active NLS. (A)** Schematic representation of the CHIKV CP NLS-EGFP fusion protein and partial sequence alignment of SFV and CHIKV capsid regions with the NLS determining sequences shaded in gray. **(B)** HEK293 cells after transfection with eucaryotic expression constructs for the CHIKV CP NLS domain in fusion with EGFP (pNLS-EGFP) or pEGFP as control. The transfected cells were analyzed by confocal laser scanning microscopy. **(C)** Graphical representation indicating the nuclear/cytoplasmic ratio of NLS-EGFP compared to cells expressing EGFP only.

**Figure 2:**
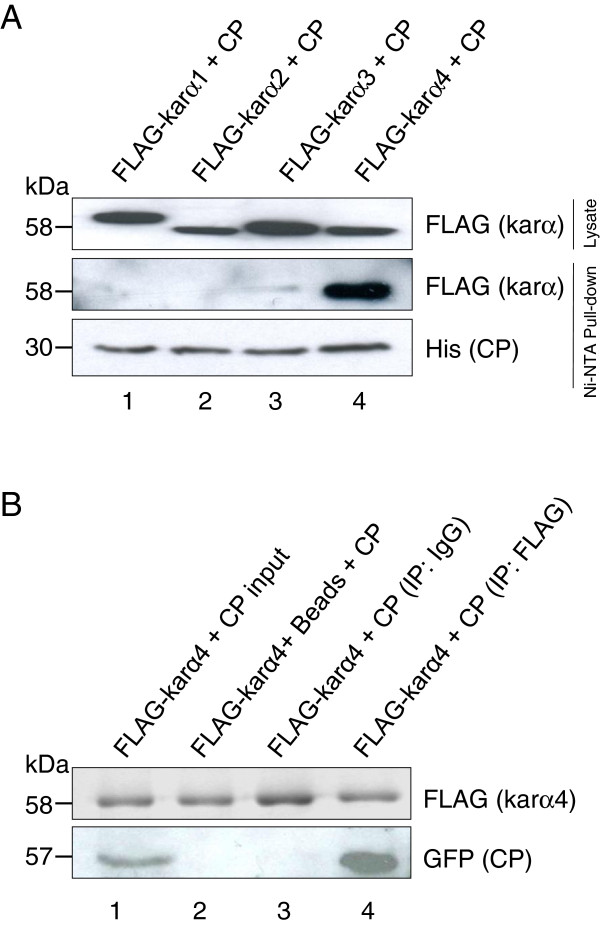
**CHIKV CP interacts with karyopherins. (A)** Ni-NTA pull-down assay with purified CHIKV CP and cell extract containing the FLAG-tagged karyopherins α1, α2, α3, and α4. Complexes consisting of karyopherin and CHIKV CP were detected with an anti-FLAG antibody. The lower panel shows the CP probed with an anti-His antibody. **(B)** Immunoprecipitations after HEK293 cells were transfected with FLAG-tagged Karα4 and pCHIKV-CP-EGFP using anti-FLAG or control antibody. The samples were subjected to SDS-PAGE and analyzed by Western blotting with an anti-GFP antibody. The upper panel shows Karα4 probed with anti-FLAG antibody.

Semliki Forest virus (SFV) is a well-studied Old World *alphavirus* and its capsid protein is reported to have an N-terminal NLS [4,15]. To validate the role of the corresponding region in CHIKV CP, the putative NLS containing sequence was cloned in fusion with EGFP and HEK293 were transfected with pNLS-EGFP. As shown in Figure 1A and B, the NLS-EGFP fusion protein was quantitatively imported to the nucleus with 5.48 times (± 1.41) higher nuclear accumulation when compared to the control EGFP expression (1.59 ± 0.42 times nuclear). As in other *alphaviruses*, a substantial amount of CP was seen in nucleoli [4]. Karyopherins are known to directly interact in the cytoplasm with proteins bearing a NLS and to carry these through nuclear pores into the nucleus. To investigate possible mechanisms of CHIKV CP nuclear translocation, we analyzed the interactions of this protein with various karyopherins. Recombinant CHIKV CP was expressed in *E. coli* as a His-tagged protein and purified using the Ni-NTA column system. HEK293 cells transiently transfected with various FLAG-tagged karyopherins (Karα1-4) were lyzed and incubated with the purified CHIKV CP. Pull-down assays revealed that the viral CP interacts strongly and specifically with Karα4 (Figure 2A, lane 4). To confirm the observed interaction between CHIKV CP and Karα4, HEK293 cells were cotransfected with FLAG-Karα4 and CHIKV-CP-EGFP. Cells were lysed and the protein complex was co-immunoprecipitated with an anti-FLAG antibody. Bound proteins were subjected to SDS-PAGE followed by Western blot with an anti-GFP antibody. As shown in Figure 2B, the band detected in lane 4 indicated that CHIKV CP co-immunoprecipitates with Karα4. The experiment was performed five times and similar results were obtained each time. No binding was observed in the control experiments using only beads or a non-specific antibody.

### The CHICK CP binds to the Karα4 major NLS binding site

**Figure 3:**
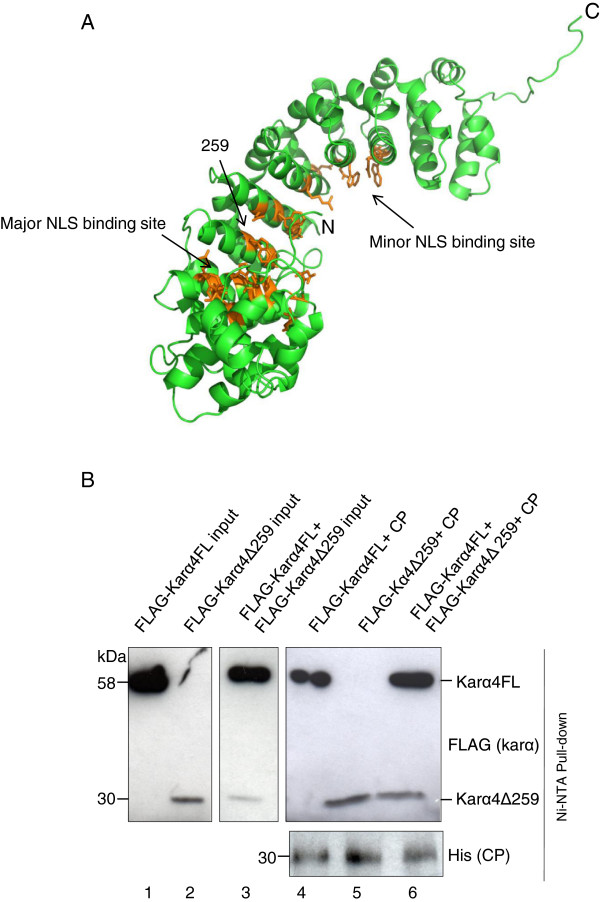
**Interaction of Karα4 NLS major binding site with CHIKV CP. (A)** Molecular model of Karα4. The residues highlighted in orange are conserved residues of Karα4 forming the surface of the major and the minor NLS recognition site. N- and C-terminus are indicated by N and C, respectively. **(B)** CP binds to the Karα4 major binding site. HEK293 cells after transfection with FLAG-tagged pCAGGSKarα4, Karα4Δ259, or both were lysed and incubated with purified CHIKV CP followed by Ni-NTA pull-down. The upper panel (lanes 1 to 6) was probed with anti-FLAG and the corresponding lanes 4 to 6 in the lower panel with an anti-His antibody.

Karα molecules contain two NLS binding sites that directly recognize NLS sequences of cargo proteins. The primary NLS binding site is located at the N-terminal arm (alpha helices that form a hairpin structure) repeats 2 to 4, while the secondary NLS binding site is located at the C-terminal arm repeats 7 to 9 (Figure 3A). The NLS major binding site is involved in the interaction with monopartite NLS, while the minor binding site associates with bipartite NLS. The acidic residues of the binding site undergo electrostatic interactions with positively charged functional groups of basic amino acids in NLS peptide regions. The hydrophobic chains of basic residues, like lysine and arginine in the NLS peptide, also form hydrophobic interactions with conserved residues of the binding site [16-19]. To further characterize the specific mechanism of CHIKV CP binding to Karα4 molecule, we created the deletion mutant Karα4Δ259, which lacks the minor binding site, and analyzed whether this region is required for the interaction with CHIKV CP by pull-down assay. Indeed, Karα4Δ259 was able to interact with CHIKV CP both alone (Figure 3B, lane 5), and in the presence of excess full-length Karα4 (Karα4-FL) protein (Figure 3B, lane 6). This demonstrates that CP binds to the Karα4 NLS major binding site. One can conclude that CHICKV CP and Karα4 binding occurs as a monopartite NLS-driven interaction.

### The CHIKV CP contains a CRM1-dependent NES

**Figure 4:**
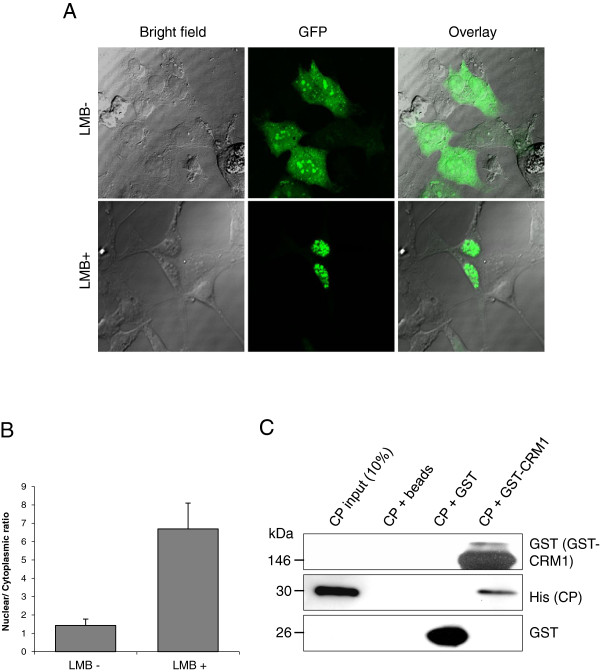
**CHIKV CP contains a CRM1-dependent NES. (A)** HEK293 cells transfected with pCHIKV-CP-EGFP were treated with LMB or mock and analyzed by confocal laser scanning microscopy. **(B)** Graphical representation indicating the nuclear/cytoplasmic ratio of and untreated cells and cells treated with LMB. **(C)** CHIKV CP directly interacts with CRM1. The pull-down assay was performed using GST-tagged CRM1 or controls with purified CHIKV CP. The latter was detected by an anti-His antibody. Upper and lower panels show GST or GST-CRM1.

To verify whether CHIKV CP is a nuclear-cytoplasmic shuttling protein, we used the specific CRM1-mediated nuclear export inhibitor leptomycin B (LMB) [20]. HEK293 cells were transfected with pCHIKV-CP-EGFP and maintained in the presence of 15 ng/ml LMB. As shown in Figure 4A and B, treatment with LMB led to an almost exclusive nuclear accumulation of CHIKV-CP-EGFP (6.68 ± 1.41 times nuclear), whereas in mock treated cells the protein remained distributed in both compartments, the nucleus and the cytoplasm (1.42 ± 0.34 times nuclear). The experiment was repeated using HeLa cells and similar results were observed (data not shown). The presence of this NES was further confirmed through GST pull-down assays. Purified CHIKV CP directly interacted with purified GST-tagged CRM1 whereas no interaction was observed in control experiments (Figure 4C).

### THE CHIKV CP NES is mapped to a leucine-rich domain

**Figure 5:**
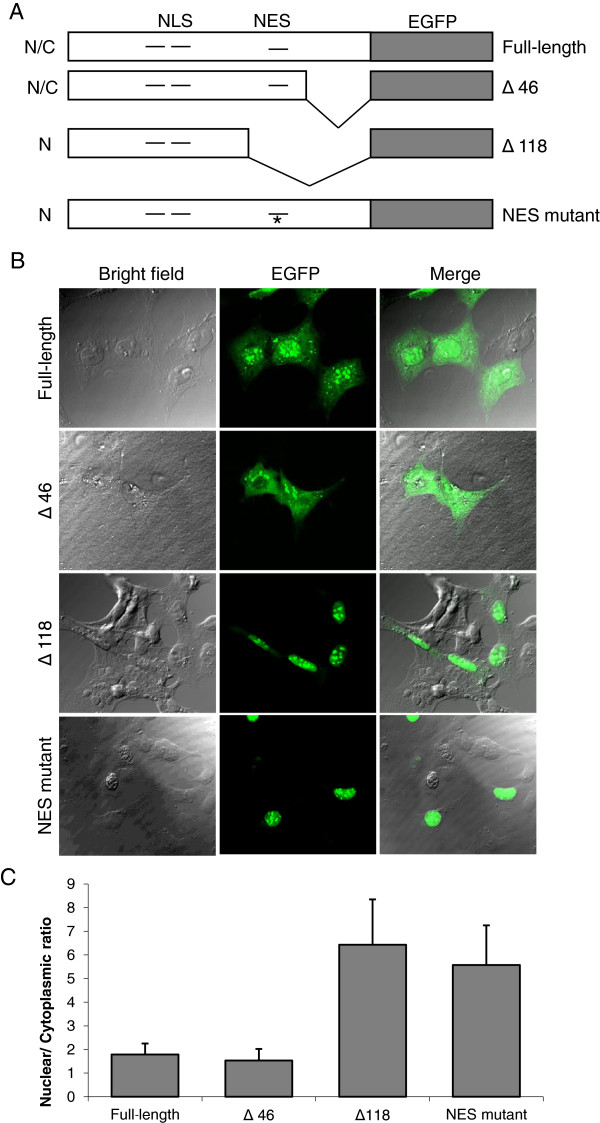
**Mapping of the NES. (A)** Various CHIK-CP-EGFP fusion constructs were used to map the nuclear export signal and their localization with N/C found in the nucleus and the cytoplasm and N in the nucleus. **(B)** HEK293 cells were transfected either with full-length pCHIKV-CP-EGFP, Δ46, Δ118, or an NES mutant (DLAKL to DAAKA indicated by asterisk (* in A) and analyzed by confocal laser scanning microscopy. **(C)** The ratio of nuclear and cytoplasmic GFP accumulation served as a determinant of nuclear import and export.

**Figure 6:**
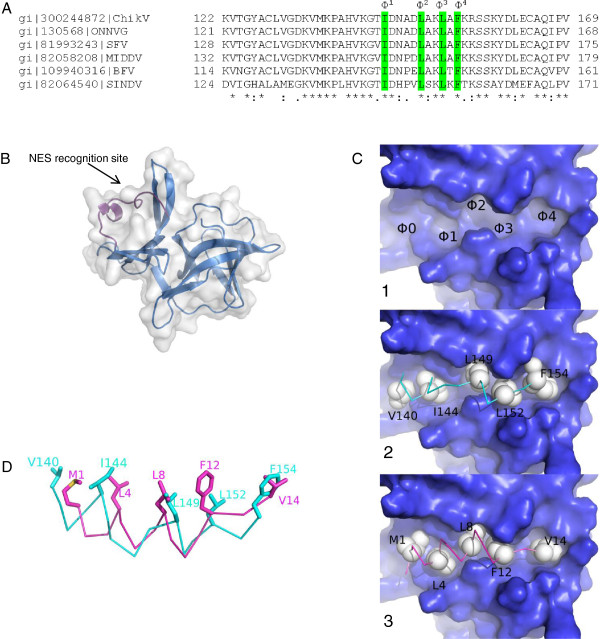
**NES conservation amongst Old World *alphavirus *isolates and structural analysis of the CHIKV CP NES. (A)** Various Old World *alphavirus* CP sequences were aligned using the ClustalW2-Multiple Sequence Alignment tool. The proposed NES is conserved amongst Old World *alphavirus* CPs (ONNVG: O’nyong-nyong virus strain G; SFV: Semliki Forest virus; MIDDV: Middelburg virus; BFV: Barmah Forest virus; SINDV: Sindbis virus). The contributing amino acids are shaded in green. Asterisks (*) indicate identical residues while colons (:) mark conserved and periods (.) semi-conserved substitutions. **(B)** The NES of the CHIKV CP forms a protrusion which is ideal for docking to proteins like CRM1. This NES region is colored magenta. **(C)***In silico* docking of NES into the CRM1 NES binding site. (1) The CRM1 binding site for NESs is shown in blue and the pockets are labeled with Φ0 to Φ4. (2) The hydrophobic residues Φ0 to Φ4 that form the NES of CHIKV CP are docked into the respective binding pockets. (3) The prototypic NES of snurportin-1 docked to CRM1 as in PDB ID 3NBY is shown for comparison. **(D)** Superimposition graphics of CHIKV CP NES with snurportin-1 NES as generated by PyMOL. Snurportin-1 is colored in magenta and CHIKV CP in cyan. The major hydrophobic residues are marked.

To identify a region of CHIKV CP that may contain a nuclear export signal, several deletion mutants were constructed (Figure 5A). Deletion of 46 aa from the C-terminus did not have any effect on the nuclear-cytoplasmic translocation process, as the truncated protein was distributed throughout the cell (Figure 5B and C). Next, the region between aa 143 and 155 that is rich in leucine residues was examined as a potential NES site. This stretch of amino acids is highly conserved among Old World *alphavirus* isolates (Figure 6A). Structural analysis revealed that this region can form a protruding ridge (Figure 6B) to make it accessible for binding to the CRM1 protein. The domain that consists of a pi-helix and a loop can be flexible in solution and thus, may fold to dock with the CRM1 binding site. A larger C-terminal deletion of 118 aa removed this potential NES and rendered the mutant EGFP fusion protein highly nuclear (6.43 ± 1.91 times more nuclear; Figure 5B and C). To further characterize the NES, two leucine residues at aa positions 149 and 152 were exchanged against alanine by means of an overlap PCR. Expression of the altered protein designated NES mutant resulted in a significant decrease of cytoplasmic localization (5.57 ± 1.67 times more nuclear; Figure 5B and C). These results provide evidence that the region between aa 149 and 152 acts as a NES that enables the CHIKV CP to be exported from the nucleus to the cytoplasm via CRM1.

### *In silico* docking of CHIKV CP NES and CRM1 supports experimental data

A structural model of docking between CHIKV CP NES and CRM1 shows the mode of binding between the molecules (Figure 6C). The molecular interaction is similar to that observed between snurportin-1 NES and CRM1 (PDB 3NBY) despite minor differences in the spacing of hydrophobic residues. The latter can also be superpositioned in the same orientation (Figure 6D) and form a consensus NES which is structurally equivalent. The modeled structure of CHIKV CP NES and snurportin-1 possesses all the hydrophobic residue side chains aligned to one face of helical structure. The comparison of binding energy between NES and CRM1 shows the global binding energy for CHIKV CP NES and CRM1 to be −71.55 joules, whereas the value for the snurportin-1 NES is −96.78. NES conserved hydrophobic residues binding to CRM1 pockets contribute most to the global binding energy (Table 1). This shows further insight to the mode of interaction between NES and CRM1 for nuclear export, and on the role of NES hydrophobic residues to dock with CRM1.

**Table 1:** **The energy contribution of hydrophobic NES residues binding to Φ**^**1**^**, Φ**^**2**^**, Φ**^**3**^**and Φ**^**4 **^**of CRM1**

**The binding pocket**	**Φ**^**1**^	**Φ**^**2**^	**Φ**^**3**^	**Φ**^**4**^
The snurportin-1 residue docking in pocket	L4	L8	F12	V14
The snurportin-1 residue’s contribution (Joules)	−20.60	−20.37	−23.28	−16.19
The CHIKV CP NES residue docking in pocket	I4	L9	L12	F14
The CHIKV-CP NES residue’s contribution (Joules)	−31.52	−13.21	−16.50	−9.34

## Discussion

The *alphavirus* genus of the *Togaviridae* family contains a number of notable human and animal pathogens. In New World *alphavirus* infected cells, CP induces apoptosis through transcription inhibition predominantly determined by either the amino-terminal fragment of the protein or by the positively charged RNA-binding domain. In Old World *alphaviruses* which include CHIKV, CP does not induce transcriptional inhibition or apoptosis [21]. Despite the localization of CHIKV CP in the cytoplasm and the nucleus, the molecular mechanism underlying its intracellular transport as well as the regulation of import and export was unknown. Although *alphavirus* CP have multiple critical functions attributed to it, some of them remain undefined. Although *alphavirus* CPs are small enough for karyopherin-independent nuclear transport [7], some were described to harbour an NLS that mediates energy dependent transport [4,15]. A replicon based study on Venezuelan Equine Encephalitis Virus (VEEV) confirms this observation for a New World *alphavirus*[5]. Even though not on nuclear transport, there are studies showing the presence of CP in the nucleus. A recent piece of work on hnRNP-K and UBQLN4 localization in CHIKV-infected cells [22] also shows CP in the nucleus and another study on the Old World and New World *alphaviruses* on transcriptional shutoff describes nuclear distribution of CP [21].

The presence of an NLS in CHIKV CP was confirmed through confocal microscopy analysis with the putative NLS region cloned in fusion with EGFP. A distorting effect of passive diffusion by NLS-EGFP with its molecular size of roughly 30 kDa was excluded with the 56 kDa full-length fusion protein CHIKV-CP-EGFP. Subsequently, we evaluated if the fusion protein interacts with karyopherins and identified Karα4 as a specific binding partner of the viral protein as observed in previous studies for other NLS containing proteins [19,23-26]. It is likely that Karα4 physically binds to CHIKV CP in the cytoplasm and subsequently interacts with importin β1 at nuclear pores followed by a Ran-GTP-dependent translocation process to the nucleus.

Karα has two NLS binding sites that directly interact with the NLS containing protein. The Karα arm repeats 2 to 4 comprise the N-terminal, and arm repeats 7 to 9 the C-terminal NLS binding site. SFV is proposed to contain two NLS in its CP 66‘KPKKKKTTKPKPKTQPKK’83 and 92‘KKKDKQADKKKKKP’105 [4] and with the NLS sequences found in CHIKV CP, 60‘KPRRNRKNKKQKQKQQAP’77 and 84‘KKQPPKKKPAQKKKKP’99 it was tempting to look for the NLS binding region in Karα. A deletion of the C-terminal NLS binding site from Karα4 did not have any effect on molecular interaction which shows that the N-terminal NLS binding site of Karα4 is sufficient for the NLS interaction. In a previous study on VEEV CP [5], a similar observation pointed towards a monopartite NLS. Together with the current data it is likely that CHIKV CP bears a monopartite NLS.

Protein export from the nucleus is mainly regulated by nuclear export receptors. CRM1 is the most common export receptor involved in the transport of many different classes of proteins [13,14]. CRM1 binding is often associated with a leucine-rich NES. As the CHIKV CP is present in both the nucleus and the cytoplasm, we investigated if CHICKV CP export is CRM1-dependent. Treatment with LMB of cells transfected with CHIKV-CP-EGFP resulted in complete inhibition of nuclear export and therefore, confirmed binding of CRM1 to CHIKV CP. Direct interaction between CRM1 and purified CHIKV CP was subsequently demonstrated through a GST pull-down assay, providing further evidence to support the role of CRM1 in CHIKV CP nuclear export.

In the VEEV CP, a NES is located in the N-terminal region which is, however, not seen in Old World *alphaviruses*[5]. To identify and characterize the NES in CHIKV CP, we tested deletion mutants fused to EGFP. Removal of 46 C-terminal aa had no effect as the protein remained in both cellular compartments. The leucine-rich region between aa 143 and 155 was further examined as a potential NES site. NES occur frequently in protein sequences, but may not be interacting with CRM1, mainly as they can be hidden within the hydrophobic core of the protein rendering them inaccessible for CRM1. Once such sequences are separated and expressed in another context, e.g. as a fusion protein, they may bind to CRM1 and promote nuclear export beyond any relevance for the respective native protein [27-29]. Structural analysis of CHIKV CP as whole protein showed that it forms a protruding ridge kind of structure, thereby making it accessible for the hydrophobic cleft of the CRM1 binding site made as to assume its biological relevance. To look at the possible role of this motif in CHIKV CP nuclear export, deletion of the 118 C-terminal aa rendered the remaining protein exclusively nuclear. Since removal of such a bulk part of the protein can largely affect its structural and functional properties, it was necessary to confirm our results by an additional method. Through site-directed mutagenesis the leucine residues at positions 149 and 152 were substituted for alanine. These alterations rendered the protein fully nuclear and thus, the CHIKV CP NES was mapped between aa 143 and 155.

Some NES are not optimized for maximum CRM1 binding [30-32] as too strong NES-CRM1 interactions would cause the Ran-free form of CRM1 to stably bind the NES in the cytoplasm and thus to re-import the protein into the nucleus. The proposed CHIKV CP NES (**I**DNAD**L**AK**L**A**F**) has spacings of hydrophobic positions Φ^1^xxxxΦ^2^xxΦ^3^xΦ^4^ (Φ=L, I, V, F, or M; x is any amino acid). This is more similar to a non-classical NES (Table 2) than the prototypical classical NES Φ-x_2-3_-Φ-x_2-3_-Φ-x-Φ. An upstream valine present in the CHIKV CP can also add to the NES (**V**KGT**I**DNAD**L**AK**L**A**F**) according to new NES consensus in which a Φ^0^ is present followed by Φ^1^xxxxΦ^2^xxΦ^3^xΦ^4^. *In silico* docking reveals that the CHIKV CP NES has a mode of binding similar to the snurportin-1 CRM1 complex, as reported in PDB ID 3NBY. The conserved hydrophobic residues of the consensus NES in snurportin-1 and CHIKV CP are protruding on one face of the structure to dock into the CRM1. The hydrophobic side chains of CHIKV CP NES can be superimposed to the respective side chains of snurportin-1. The snurportin-1 and CHIKV CP have differences in the number of spacings between conserved hydrophobic residues, but these are accommodated with minor conformational deviations in the backbone whereas the conserved hydrophobic side chains in two structures occupy equivalent space, respectively. Even though suboptimal spacing can weaken CRM1 binding like that of snurportin-1 [27], the difference in spacing should not affect docking itself, as NES ligands can compensate for different Φ spacings by adapting their conformations to the rigid NES binding site. This variability in Φ spacing can cause greater diversity in functional NES as all hydrophobic positions are not necessarily essential if other Φ positions are strong [27]. Here, hydrophobic NES residues including leucines at 149 and 152 contribute strongly to the NES binding site as seen with their binding energy, or as mutating those to alanine inhibited the nuclear export.

**Table 2:** Previously described non-classical NES sequences

**Organism**	**Protein**	**Sequence**	**Reference**
Mouse mammary tumor virus	Rem	**L**T**L**F**L**A**LL**SV**L**	[33,34]
Human endogenous retrovirus	Rec	**W**AQ**L**KK**L**TQ**L**	[33]
Equine infectious anemia virus	Rev	P**L**ESDQ**W**CRV**L**RQS**L**	[33,35]
Severe acute respiratory syndrome corona virus	9b	**L**R**L**GSQ**L**S**L**	[36]
Rous sarcoma virus	Gag	**L**TD**W**AR**V**REE**L**	[37]
Hepatitis C virus	Core	**L**GKV**I**DT**L**TCGFAD**L**	[38]
Homo sapiens	P27	**L**FGPVDHEE**L**TRD**L**	[39]
Homo sapiens	Focal adhesion kinase	**L**RSEE**V**HW**L**H**V**D**M** (NES1)	[40]
		**L**D**L**AS**L**I**L** (NES2)	
African swine fever virus	P37	**L**T**V**EE**L**G**L** (NES1)	[41]
		**I**DS**I**QT**V**QQ**M** (NES2)	

Amino acids important for NES function are bold.

Nuclear cytoplasmic shuttling of viral proteins was reported to be for instance associated clinical severity. Lesser pathogenic VEEV strains contain a wide variety of mutations in the CP that affect its inhibitory function in nuclear import. Therefore, these mutations appear to be the determinants of this attenuated phenotype. Mutagenic analysis of the linker peptide between NES and NLS has demonstrated that modifications in the linker peptide of VEEV CP attenuated the ability of H68 peptide to inhibit nuclear import [5]. *Alphaviruses* have characterized NLS in both NSP2 and CP: In Old World *alphaviruses* NSP2 but not CP induces apoptosis [21,42-44], while in New World *alphavirus* apoptosis is induced by CP [45] and not NSP2 [21]. This also means that both, Old and New World *alphaviruses* have different functions associated with its nuclear transport of proteins. The fact that *alphaviruses* replicate in enucleated vertebrate [46] but not invertebrate cells [47] points to a different role of nuclear transport in both cell types. It may be possible that nuclear transport of CP is important if not essential in invertebrates like mosquito as the alternate host but not in vertebrates. Comparing the replication of Chikungunya virus in both cell types with and without inhibiting nuclear import and export may help to clarify the differences. At this stage, the exact role associated with CHIKV CP nuclear import and export remains unclear and has to be further investigated in an infectious system.

## Conclusions

In summary, we could show in a virus-free system that the CHIKV capsid protein exhibits both a NLS and a NES. CHIKV CP is actively transported between the cytoplasma and the nucleus. We conclude that this protein has the ability to shuttle via interaction with karyopherins for its nuclear import and, *vice versa*, by CRM1-dependent nuclear export.

## Methods

### Plasmids, cloning, and mutagenesis

The Chik-Wü virus isolate (Genbank: EU037962) or subclones thereof (GenBank: HM369441) served as templates for PCR amplification of the CHIKV capsid and all subclones [3,48]. To clone pCHIKV-CP-EGFP, full-length CHIKV CP was amplified from our previously described expression plasmid in which the protease cleavage site had been inactivated by exchanging serine 213 to alanine [3]. The amplimer created with the oligonucleotide primers HindIIIChikcap_fwd 5′-*aagctt*atggagttcatcccaacccaaac-3′ and ChikcapXmaI_rev 5′-at*cccggg*tccactcttcggctc-3′ was inserted into pEGFP-N1 (Clontech) into the restriction sites *Hind*III and *Xma*I in frame with EGFP. The NLS region in CP located between aa positions 60 and 100 was inserted into pEGFP-N1 after amplification with primers HindIIINLScap_fwd 5′-*aagctt*atgaagccacgcaggaatcgg-3′ and XmaINLScap_rev 5′-at*cccggg*tcggcttctttttcttttg-3′ to create pNLS-EGFP. Construction of Chikcap Δ46 in which 46 aa were deleted from the C-terminus has been described previously [3]. The mutant with the larger C-terminal deletion Δ118 was constructed with HindIIIChikcap_fwd and Chikcap118_rev 5′-at*cccggg*tcccctttacgtgtgctgg-3′. To mutate leucines of CP at position 149 and 152 to alanine, an overlap PCR was performed with the above and overlapping primers 5′-cttaaaggccgctttggccgcgtccgc-3′ and 5′-gcggacgcggccaaagcggcctttaag-3′. Expression in *E. coli* of the complete CHIKV CP was carried out by inserting a PCR-derived product amplified with chk_petcapsid_fwd 5′-*ggatcc*atggagttcatcccaacc-3′ and chk_petcapsid_rev 5′-*ctcgag*tccactcttcggctc-3′ from pChikcap [3] into pET28a (Invitrogen) between *BamH*I and *Xho*I sites. pGEX-CRM1 containing GST-tagged CRM1 was a kind gift of Nabeel R. Yaseen [49]. The mammalian expression vectors pCAGGS containing either Karα1, 2, 3, or 4 with N-terminal FLAG-tags were a generous gift of P. Palese and have been described previously [50,51]. The deletion mutant pCAGGS Δ259 Karα4 was constructed by digesting the full-length vector with *Tth*111I followed by Klenow filling and religation.

### Protein expression and purification

*Bl21* bacterial cells (Invitrogen) were transformed with N-terminally His-tagged pet28aCHIKV CP and allowed to grow in 500 ml LB medium with kanamycin. The culture was grown at 37°C until OD600 had reached 0.6, induced with IPTG at a concentration of 1 mM, and kept overnight at 21°C in a rotary shaker. The cells were pelleted and resuspended in 6 ml of buffer containing PBS and 5% glycerol, and lysed by sonication. The cell lysate was spun at 5,000 rpm for 20 min and imidazole was added to the supernatant at a concentration of 10 mM. Precharged and prewashed Ni-NTA beads (Invitrogen) of 0.6 ml of bed volume were mixed with the cell lysate at 4°C for 1 h, loaded onto an affinity column, and washed with 50 ml of PBS containing 20 mM imidazole and 5% glycerol. CP was eluted using elution PBS with 5% glycerol and 300 mM imidazole. Eluents were dialyzed against buffer containing PBS and 5% glycerol followed by confirmation by SDS-PAGE and Western blot.

### Pull-down assay

HEK293 cells untransfected or transfected with either of the FLAG-tagged constructs pCAGGSKarα 1, 2, 3, 4, or Δ259Karα4 were lyzed with RIPA buffer (Thermo Scientific) and incubated after adding purified His-tagged CHIKV CP. Ten μl of resuspended Ni-NTA beads were added to bacterially expressed CHIKV CP, mixed, and incubated further at 4°C. Samples were centrifuged and the supernatant was discarded. The beads were washed with RIPA buffer and boiled for 10 min after adding 40 μl Laemmli buffer. The samples were subjected to SDS-PAGE and transferred to nitrocellulose membranes. The membranes were probed with monoclonal anti-FLAG antibodies (Sigma) followed by anti-mouse secondary antibodies conjugated with horseradish peroxidase (Santa Cruz Biotechnology). Bands were visualized with enhanced chemiluminescence detection reagents (Amersham). GST pull-down assay was performed using glutathione-Sepharose resin (GE Healthcare) according to manufacturer’s instructions. Briefly, GST or GST-CRM1 protein immobilized on glutathione-Sepharose was incubated with 2 μg of purified CHIKV CP at 4°C overnight. Bound proteins were eluted by boiling the Sepharose in Laemmli buffer, resolved by a 12% SDS-PAGE, transferred to nitrocellulose, and analyzed by Western blot.

### Co-immunoprecipitiation

For Co-IPs, HEK293 grown on Petri dishes (10 cm) were transfected with FLAG-tagged pCAGGSKarα4 and pCHIKV-CP-EGFP using the Turbofect (Fermentas) transfection reagent according to manufacturer’s instructions. Cell lysates were prepared 48 h after transfection in 1.5 ml of RIPA buffer. Aliquots of 500 μg total protein were incubated with 1 μg of mouse anti-FLAG antibodies or control IgG for 1 h at 4°C. A 50% suspension of protein AG agarose (Santa Cruz Biotechnology) was added to the lysate and incubated for 2 h at 4°C while shaking. The agarose beads were collected by centrifugation and washed by three with RIPA buffer. After the final wash, the supernatant was discarded and the beads were resuspended in Laemmli buffer and boiled for 10 min. The immunoprecipitated material was subjected to 12% SDS-PAGE and analyzed by Western blot using an anti-GFP antibody.

### Transfection, treatment with LMB, and fluorescence microscopy

HEK293 cells were maintained in DMEM supplemented with 10% FCS, 100 U/ml penicillin, and 100 μg/ml streptomycin. HEK293 seeded in 12-well plates at a density of 2 × 10^5^ per well were transfected with Turbofect (Fermantas). For the experiments with LMB, cells were treated with LMB at a concentration of 15 ng/ml for 6 h. To detect EGFP-tagged proteins by fluorescence microscopy, the cells were fixed for 10 min in PBS with 4% formaldehyde, washed twice in PBS, and mounted on slides with an anti-fade mounting media (Dako). The slides were analyzed on a Leica confocal microscope and cells with moderate GFP expression were selected for analysis.

### Nuclear localization analysis

NIH Image J v1.30 software was used to quantify the nuclear localization of GFP fusion proteins as described before [52]. All confocal images were quantified at settings where the intensity of GFP fluorescence ranged up to 250 pixel values. To examine the nuclear localization of the GFP fusion proteins, a square with an area of 225 pixels was used to measure the mean intensities of six different areas in the nucleus and the cytoplasm of a representative cell from each of three transfections. Thus, eight representative cells analyzed yielded a total of 48 areas from each, the nucleus and the cytoplasm. Relative nuclear localization is reported as the ratio of the mean intensity of GFP fluorescence in the nucleus and cytoplasm (nuclear/cytoplasmic ratio, N/C ratio).

### Sequence alignment, protein modelling, and *in silico* protein-protein docking

Various Old World *alphavirus* CP amino acid sequences were aligned with the ClustalW2 - Multiple Sequence Alignment tool http://www.ebi.ac.uk/Tools/msa/clustalw2/. The structures of CHIKV CP and Karα4 were modeled using the automated homology modelling server 3D-JIGSAW [53] which used PDB ID 1VCP and 1BK6, respectively, as template. Figures were generated using PyMOL: http://www.pymol.org. To perform *in silico* protein-protein docking, the NES region of CHIKV CP was modeled using the loop building tool of the SPDBV [54]. The hydrophobic conserved CHIKV CP NES residues were kept positioned in the NES binding site and superimposed to the respective NES residues as in chain A of PDB ID 3NBY followed by energy minimization. The docking was further optimized by a flexible docking method using flex dock server [55]. The energetically most favorable structure was used for the CHIKV CP NES and CRM1 interaction analysis and binding energy was calculated using firedock server [56,57].

## Abbreviations

CHIKV: Chikungunya virus; CP: Capsid protein; NLS: Nuclear localization signal; NES: Nuclear export signal; CRM1: Chromosomal region maintenance 1; NPC: Nuclear pore complex; Karα: Karyopherin α; LMB: Leptomycin B; VEEV: Venezuelan equine encephalitis virus; PDB: Protein data bank.

## Competing interests

The authors declared that they have no competing interest.

## Authors’ contributions

ST designed and performed experiments, analyzed data and wrote the manuscript. JR performed *in silico* experiments. LJ analyzed data. SS designed experiments and analyzed data. BMP and OH analyzed data and edited the manuscript. All authors read and approved the manuscript.
